# “It is important to consult” a linguist: Verb-Argument Constructions in ChatGPT and human experts’ medical and financial advice

**DOI:** 10.1371/journal.pone.0324611

**Published:** 2025-05-27

**Authors:** J. Elliott Casal, Christopher M. Stewart, Alistair J. Windsor

**Affiliations:** 1 Department of English, University of Memphis, Memphis, Tennessee, United States of America; 2 Institute for Intelligent Systems, University of Memphis, Memphis, Tennessee, United States of America; 3 Department of Mathematical Sciences, University of Memphis, Memphis, Tennessee, United States of America; University of Konstanz: Universitat Konstanz, GERMANY

## Abstract

This paper adopts a Usage-Based Construction Grammar perspective to compare human- and AI-generated language, focusing on Verb-Argument Constructions (VACs) as a lens for analysis. Specifically, we examine solicited advice texts in two domains—Finance and Medicine—produced by humans and ChatGPT across different GPT models (3.5, 4, and 4o) and interfaces (3.5 Web vs. 3.5 API). Our findings reveal broad consistency in the frequency and distribution of the most common VACs across human- and AI-generated texts, though ChatGPT exhibits a slightly higher reliance on the most frequent constructions. A closer examination of the verbs occupying these constructions uncovers significant differences in the meanings conveyed, with a notable growth away from human-like language production in macro level perspectives (e.g., length) and towards humanlike verb-VAC patterns with newer models. These results underscore the potential of VACs as a powerful tool for analyzing AI-generated language and tracking its evolution over time.

## Introduction

In this paper, we compare language patterns in text from LLM-powered chatbot products (henceforth “AI-generated text”) to those found in human writing (henceforth “human-written text”). Much of the research comparing these two concerns research ethics and academic integrity (e.g., [1–4]) or broader issues of societal change or misuse (e.g., [5–9]). There is some ambiguity in this literature over the degree to which AI-generated text and human writing can be differentiated. Obviously, the commercial success of tools like ChatGPT, Gemini, and Claude is premised on a close resemblance, and some published research suggests this is true in certain ways. For example, Casal and Kessler [1], found that AI-generated research article abstracts in Applied Linguistics based on the content of a research paper were not reliably distinguishable from the published, human-composed abstracts by researchers in the field. In a different manner, Gorenz and Schwarz [10] found that humans rated human and AI-generated satirical joke headlines (based on *The Onion*) to be similarly funny, with some AI-generated jokes performing even better than human jokes. These works suggest that differentiating AI-generated text from human-written text can be challenging in at least some cases, in spite of human’s potential for creativity [11].

In contrast, research on signals that allow for the detection of AI-generated text suggests that differentiating between the two may actually be straightforward. This research generally consists of two kinds of approaches: “black-box” methods that rely on linguistic or statistical patterns in text and “white-box” methods that require access to the language model for techniques like watermarking [12]. A huge variety of signals have been used in published studies of black-box methods, including lexico-grammatical [5] and stylistic or macro-structural features [6]. While linguistic patterns “... serve as valuable features for detecting LLM-generated text”, it appears that black-box methods “will gradually become less viable as language model capabilities advance and ultimately become infeasible” [9]. Recent innovations like “SynthID-Text” [13] suggest that efficient, accurate and low-latency watermarking may soon become commonplace. These may further enhance the detectability of AI-generated text.

While these two currents may appear contradictory, we suggest that AI-generated text both accurately mimics some communicative aspects of human-written text *and* differs from it in readily detectable ways when responding to brief prompts. Berber Sardinha [5] uses brief prompts, such as “write a conversation between people of about 1000 words in length,” such that the vast majority of the final context is generated text. In comparison, Casal and Kessler [1] use a complicated prompting scheme that involves using the AI to summarize various sections of the article and then combining these summaries into a prompt that asks for an AI-generated abstract with particular parameters. In this scheme the generated text makes up a much smaller portion of the final context. The LLM-powered chatbots rely on contextualized co-occurrence probabilities of tokens, which are likely to resonate with collocational, colligational, and other phraseological relationships found in contextualized human language. This allows the models to mimic formal and, to a lesser degree, functional linguistic competence [14] by imitating human language production. Sometimes this is achieved by reproducing chunks of models’ training data [15]. In most cases, this involves conforming to very specific goals, such as the instantiation of a goal to “follow the user’s instructions helpfully and safely” via instruction tuning and reinforcement learning with human feedback [16].

From a theoretical linguistic point of view, this perspective aligns better with models that emphasize the role of phraseological relationships in representing semantic information than those which separate lexicon and grammar into separate systems. That is, explaining the similarity of AI-generated and human-written texts is straightforward under a Construction Grammar (CxG) perspective, which theorizes language knowledge as a series of form-meaning mappings generalized over time through use. In this framework, the schematic meanings of constructions are theorized as being meaningful in their own right, reflecting regularities in language use patterns. Though the local selection of tokens or words by models like ChatGPT may not match the choices of humans, if they are based on actual language usage, they will be likely to resonate with broader constructional patterns. Accounting for such patterns is not straightforward in linguistic theories that downplay the role of experience and usage with language, instead emphasizing innate knowledge and a strict boundary between lexical items and syntactic rules. While research showing that transformer-based language models contain representations of construction-like verbal patterns (e.g., [17]) suggests that LLMs can ‘tap into’ these regularities, there is a dearth of research on how LLM-powered chatbots produce such constructions. This would allow us to better understand how humans and LLM-powered chatbots use language to achieve different ends.

In this paper we adopt a Usage-Based Construction Grammar perspective, particularly focusing on Verb-Argument Constructions, to compare human and AI-generated language. To fully explore the topic, this comparison includes three GPT models (3.5, 4, and 4o) and, for GPT 3.5, two interfaces (Web vs. API) in a single broad genre (providing solicited advice) across two domains (Finance and Medicine). We use a widely used tool to identify Verb-Argument Constructions statistically in the data and probe the verbs which occupy the most frequent of such constructions to compare human text to AI-generated text on matched queries.

## Literature review

### The language of large language models

The majority of research on AI-generated language focuses on lexico-semantic differences with text written by humans. These studies have found, for instance, that AI-generated text tends to have more positive or neutral sentiment [7,9,18] and show less variable discourse markers [19]. Other studies have shown that AI-generated text contains more passive voice [20] and longer sentences with less variable length [6] in comparison to human writing. These studies offer some evidence of how AI-generated text differs from human-produced language, but they do not provide concrete insights into the extent to which AI-generated texts resonate with human conceptions of the meaning potentials associated with linguistic resources. Much of the research involves very short prompts, which contrasts with the mega prompts that are currently favored in real world applications [21].

In a step towards this goal, an emerging body of research has investigated the capacity of LLMs to re-produce context-appropriate language behaviors. One example is communicative competence, the language behaviors that humans use to achieve particular goals in different interactive contexts. For example, a speaker may say “it’s cold in here” with the intent of getting a listener to turn on the heat or close a window. A response like “Yes” with no further action may suggest linguistic competence, but in some cultural contexts it misses the more subtle intent of the speaker that a communicatively competent hearer will likely notice. The ability of LLMs to respond to such instances of “conversational implicature” was assessed in [22]. Testing different foundation models, tunings and prompting strategies showed that while LLM-powered chatbots were generally poor at resolving implicature, fine-tuning via instructions dramatically improved performance.

More directly, there is evidence for construction-like verb-argument structures in the representations stored in transformer-based language models [17,23,24]. These studies argue that such evidence lends credence to the view that lexico-semantic representations alone are inadequate for capturing all the information stored in LLMs and, by extension, the human language capacity. These constructions range in frequency from common constructions that occur in sentences like “Rita passed the note to the teacher,” to much rarer, more idiomatic constructions like the English Article + Adjective + Numeral + Noun (AANN), (e.g., “a discerning several thousand judgements”; “a lovely five days”) [25]. The models are even able to productively encode constructions that only occur a handful of times in a corpus of billions of tokens [26]. We discuss a particular type of constructions which play a prominent role in human language in detail in the next section.

Overall, this research provides an intriguing glimpse into a similarity between language representations in LLMs and human language production. It also raises the question of how LLM-powered chatbots produce the constructions captured in these representations when responding to user queries at scale. This is a difficult question to approach for several reasons. Perhaps most importantly, the output of these applications may involve additional pre- and post- processing steps that obscure what input the underlying model receives and, possibly, what output it produces. Furthermore, the underlying models have undergone alignment, which typically involves both supervised finetuning (additional training on curated language data), and often reinforcement learning from human feedback, the last of which explicitly shifts output away from that of a strict language model.

### Verb-Argument constructions

Usage-based theories of language posit that language use is the result of context-dependent cognitive processing and that linguistic knowledge is a set of form-meaning mappings that emerge and coalesce over time though use. These form-meaning mappings, referred to as constructions, range from sequences of morphemes to broader, more schematic structures. They are theorized as meaningful units in and of themselves, and form the core of human language in Construction Grammar [27,28]. Humans learn constructions over time by encountering and using them for particular functional potentials in specific social contexts. In this sense, constructions are built up from and anchored to words in constructional contexts in use. Therefore, a usage-based CxG approach to linguistics does not attempt to sever lexicon from grammar, as other linguistic theories do. Rather, CxG posits a continuum of constructions with varying degrees of complexity.

Verb-Argument Constructions (VACs) focus on verbs and their corresponding arguments in usage-based constructions. Common examples include more abstract constructions, such as the transitive ([verb][direct object]) and ditransitive ([verb][indirect object][direct object]), as well as more narrowly defined constructions, such as ‘[verb] across [noun phrase]’. As an illustration of the form-meaning mappings at the core of these constructions, a ditransitive construction has a core meaning of transfer of an object that is both built up from and reinforced through recurring instances of the construction in usage. Simultaneously, the meaning that is sedimented in the VAC allows for emergent production and interpretation of novel forms in context. Thus, a meaning can be deduced from the sentence ‘Paula crutched Tony an apple’ (example based on [29]), where the verb “inherits its interpretation from the echoes of the verbs that occupy this VAC” [30].

Considerable research has been conducted on first and second language VAC knowledge and learning (e.g., [31–40]). Other research has provided key insights into the potential psychological reality of VACs through corpus-based and experimental evidence of verb-construction associations and online construction meaning access (e.g., [35,41–43]). Findings related to VAC characteristics highlight that the most frequent verbs in many VACs are semantically somewhat generic and highly predictable, but that strong associations between VACs and lower frequency verbs also represent prominent communicative resources [31,33]. From a learning perspective, second language learners at lower levels of proficiency demonstrate reliance on more prototypical verbs in high frequency VACs, with more proficient learners demonstrating more dynamic and varied use of such resources [36]. Together, this scholarship provides evidence that VACs are psychologically real for both first and second language users.

Importantly, this research also resonates with broader usage-based claims that linguistic knowledge develops through sensitivity to frequency, contingency, and formulaicity in input [32,38]. In this sense, contextual aspects of usage (such as genre and community) are essential components of an individuals’ knowledge of a construction, as constructional knowledge reflects a language user’s linguistic experiences, which are always embedded within a social situation. To probe the context-sensitivity of VACs and VAC profiles, Casal et al. [44] extended Ellis et al.’s [33] analysis of VACs in the British National Corpus by comparing general domain usage patterns to usage in an academic research corpus and disciplinary sub corpora. Casal et al. [44] found notable consistency in the VACs themselves used across general domain and academic English contexts and across disciplines, but considerable variation in the verbs used within such constructions. That is, the language produced across contexts demonstrated consistent use of core schematic VAC resources, but the local level instantiations of these VACs were context specific. Once more, this underscores the central role of VACs in human language, as well as the prominent effect of context and purpose on linguistic choices.

### The present study

The present study compares VACs in AI-produced to human-written text on matched queries, adding a usage-based lens of specific language behaviors to the existing literature. Theoretically, VACs simultaneously capture *consistency* through the recurrence of schematic frames and prototypical forms and *variation* through the productivity of such constructional resources in usage. This affords an important window into both formal and functional patterns of language across both AI-generated and human written text.

At the same time, given what empirical research suggests regarding constructions, VAC distributions and instantiations are highly contextual, reflecting the communicative demands, expectations, and conventions of community genre practices. Thus, a VAC perspective affords researchers windows into how effectively AI-based sources of language vary their production based on purpose, topic, and domain. To our knowledge, this is the first study comparing VACs in AI-generated vs. human-written text on matched prompts, as well as the first to trace changes in AI-generated language across models.

RQ1: To what extent do frequency ranks and distributions of Verb-Argument Constructions in AI-generated text in the Medicine and Finance domains reflect the language produced by humans in similar tasks?

RQ2: To what extent do the verb frequency ranks and distributions of the top Verb-Argument Constructions in AI-generated texts in these domains reflect the language produced by humans in similar tasks?

## Methods

### The corpus

The materials used for these analyses are a subset of the Human ChatGPT Comparison Corpus HC3 [7], which contains “nearly 40K questions and their corresponding answers from humans and ChatGPT covering… open-domain, computer science, Finance, Medicine, law, and psychology” (p. 2). It was compiled when GPT3.5 was the LLM powering ChatGPT. Question-answer pairs from human experts were taken from publicly available datasets and Wikitext. To obtain answers to these same questions from ChatGPT, the authors presented the same questions manually via OpenAI’s site. While HC3 has both English and Chinese-language data, we only draw from the English language data. Our analysis includes language from the Finance and Medical domains. Our analysis was conducted on a version of the corpus we accessed on May 10, 2024. Individual participants were not identifiable based on the information present in the human responses.

The corpus required some cleaning due to the presence of system-level responses, e.g., “too many requests in 1 hour”, and multiple AI-generated responses for some queries. We eliminated the system level responses and only included the first ChatGPT response in instances with multiple entries. This yields 3,932 question-answer pairs (human and ChatGPT web) in the Finance domain and 1,237 question-answer pairs (human and ChatGPT web) in the Medical domain. The questions were diverse, but we provide examples here. One relatively short Finance question is: “My medical bill went to a collection agency. Can I pay it directly to the hospital?” The Medical domain questions often contained more narrative details and structure, for example: “What could it be if child is having intermittent cough inspite of taking medication? 18 month old boy has a recurring dry cough, mainly at night. It will last 3-4 days then loosen and disappear only to return about 5-7 days later. I have tried everything including vaporizers, humidifiers, vaporub, glycerin and honey etc. What could this be?!”

We then presented the same question text to ChatGPT 3.5, GPT-4 and GPT-4o via the OpenAI API. We set the temperature to 0.7, the OpenAI “default” and the suspected temperature setting for the web interface. We therefore have two responses from ChatGPT 3.5, which differ in the way that the question text was presented to the model. We hypothesize that queries that use the web interface get extensive preprocessing before being presented to the model. Moreover, though the original paper did not note the particular variant of the GPT-3.5 model that was used, we are certain that the variant of the GPT-3.5 (gpt-3.5-turbo-0125) that we used for our GPT-3.5 API corpus is newer than that used for the GPT-3.5 Web. The cleaned and expanded dataset can be accessed via the Harvard Dataverse through Stewart, Windsor, and Casal [45].

Tables 1–3 provide the summary statistics for the two corpora. Table 1 summarizes the questions across Medicine and Finance domains. Table 2 provides information about the Medicine responses and Table 3 does the same for the Finance responses.

**Table 1:** Summary statistics for questions.

	Medicine		Finance	
	Mean	SD	Mean	SD
**Question words**	75.4	14.4	12.1	5.0
**Question sentences**	4.0	2.1	1.1	0.3
**Question words per sentence**	26.1	19.5	11.5	4.7

**Table 2:** Summary statistics for the Medical Corpus responses.

	Mean	SD	Mean diff	SD diff	*p*-value
**Human words**	92.4	51.6			
**ChatGPT 3.5 Web words**	213.3	60.0	121.0	75.8	0.0000
**ChatGPT 3.5 API words**	202.4	73.6	110.0	89.3	0.0000
**ChatGPT 4 words**	401.6	101.2	309.2	111.1	0.0000
**ChatGPT 4o words**	461.7	115.5	369.3	124.1	0.0000
**Human sentences**	5.2	3.7			
**ChatGPT 3.5 Web sentences**	8.9	2.9	3.8	4.6	0.0000
**ChatGPT 3.5 API sentences**	10.1	5.3	4.9	6.5	0.0000
**ChatGPT 4 sentences**	20.6	6.5	15.5	7.5	0.0000
**ChatGPT 4o sentences**	25.8	7.8	20.7	8.6	0.0000
**Human words per sentence (WPS)**	26.7	23.4			
**ChatGPT 3.5 Web WPS**	24.4	4.1	-2.3	23.6	0.0006
**ChatGPT 3.5 API WPS**	22.0	5.0	-4.7	24.1	0.0000
**ChatGPT 4 WPS**	20.2	3.6	-6.5	23.7	0.0000
**ChatGPT 4o WPS**	18.4	2.9	-8.3	23.7	0.0000

Note: all comparisons made to the human corpus

**Table 3:** Summary statistics for the finance corpus responses.

	Mean	SD	Mean diff	SD Diff	*p*-value
**Human words**	202.0	185.4			
**ChatGPT 3.5 Web words**	232.3	72.9	30.4	192.3	0.0000
**ChatGPT 3.5 API words**	188.9	98.7	-13.1	200.1	0.0000
**ChatGPT 4 words**	464.9	160.5	262.9	232.4	0.0000
**ChatGPT 4o words**	538.9	181.7	337.0	246.6	0.0000
**Human sentences**	9.0	8.1			
**ChatGPT 3.5 Web sentences**	8.5	2.9	-0.5	8.3	0.0002
**ChatGPT 3.5 API sentences**	9.3	6.3	0.3	9.7	0.0585
**ChatGPT 4 sentences**	22.8	8.9	13.8	11.3	0.0000
**ChatGPT 4o sentences**	27.9	10.8	18.9	12.8	0.0000
**Human Words per Sentence (WPS)**	22.9	8.5			
**ChatGPT 3.5 Web WPS**	28.1	8.0	5.1	11.6	0.0000
**ChatGPT 3.5 API WPS**	23.2	7.7	0.3	11.3	0.1463
**ChatGPT 4 WPS**	21.4	4.9	-1.6	9.6	0.0000
**ChatGPT 4o WPS**	20.3	5.6	-2.7	9.9	0.0000

Note: all comparisons made to the human corpus

We note that the questions are significantly longer in the Medical domain, with both more and longer sentences. In contrast, questions in the Finance domain are typically a single short sentence. Therefore, comparisons across domains are not a focus of the analysis.

In Guo et al.’s [7] analysis of the language patterns in AI-generated text and human experts in this corpus, they noted that it was easier to identify text output by ChatGPT when naïve raters could compare it to a similar text written by a human expert. They observe that although raters generally found answers from ChatGPT to be more “helpful”, this was not the case in the Medical domain, which the authors hypothesized was due to the ChatGPT’s preference for lengthy responses in contrast to Medical professionals’ more succinct, straightforward answers. The text written by experts was less literal in its interpretation of the question and showed more individuality than output by ChatGPT.

When comparisons across models and interfaces (API vs. WEB) are made (see Table 4), we see that responses are dramatically longer for GPT-4 and GPT-4o than for GPT-3.5. GPT-4o is the most verbose though it favors slightly shorter sentences than GPT-4. The differences within GPT responses are consistent across the two corpora and all are significant at the 0.001 level in a one-sided paired sample t-test.

**Table 4:** Model and interface rank order by verbosity metrics.

Words:	3.5 API < 3.5 Web < 4 < 4o
Sentences:	3.5 Web < 3.5 API < 4 < 4o
Words per Sentence:	4o < 4 < 3.5 API < 3.5 Web

### Verb-Argument construction analysis

Our goal is a descriptive and comparative analysis across AI-generated and human-written text in two domains of discourse: Finance and Medicine. Verb-Argument Construction types, frequencies, and variants were identified automatically using the Tool for the Automatic Analysis of Syntactic Sophistication and Complexity 1.3.8 [46]. TAASSC is a freely available text complexity analyzer that includes traditional holistic and global measure of syntactic complexity (e.g., length of production unit measures), fine-grained measures of both phrasal and clausal complexity, and a number of measures aligned with usage-based approaches to language including those associated with VACs. In computing VAC-based indices in relation to VAC profiles in the Corpus of Contemporary American English [47], TAASSC generates “clause database” files with each finite clause labeled with a VAC and the lemmatized verb occupying the verb slot indicated. The authors used a modified version of TAASSC that indicated both verb and lemmatized verb (the modification is only necessary for the ConcordanceCompass tool). These “clause database” files were combined and tallied to produce a summary that listed each VAC’s proportion and raw frequency, as well as a lemmatized verb list for each VAC targeted for follow-up analysis. Identified VACs in the TAASSC output were not further collapsed or manually grouped in any way.

## Results

### Verb-Argument constructions across subcorpora

Table 5 presents the top VACs analyzed in this paper with definitions and examples drawn from human-produced and ChatGPT-generated text. Tables 6 and 7 present the top ten VACs extracted from prompt responses to ChatGPT and written by humans across Financial and Medical domains of discourse. Also presented is the percent of overall VACs that each VAC represents in the corresponding subcorpus. There were at least 250 VAC types which occurred 5 times or more in each subcorpus for the Medicine domain and 759 which met this condition in the Finance domain. VAC types were not combined or modified from the TAASSC output.

**Table 5:** Top VACs defined and exemplified in Human writing and LLM-powered chatbot text.

VAC Label	VAC Description	Human	GPT
*dep-nsubj-v-dobj*	Dependent - Subject - Verb - Direct Object	Hope I have answered your question. (Med)	Capital gains are taxable, and the rate at which they are taxed depends on how long you held the stock. (Fin 3.5)*
*mark-nsubj-vcop-acomp*	Subordinator - Subject - Copula Verb - Adjective Complement	It makes no sense to do this **if the value of your asset is static**. (Fin)	**If the interest rates on savings accounts are lower** than the rate of inflation, the real value of saved money decreases over time. (Fin 4)
*mark-nsubj-v-dobj*	Subordinator - Subject - Verb - Direct Object	Make sure **that you include every minute detail possible**. (Med)	**Since you have a dust allergy,** antihistamines might help manage your allergic symptoms, which can exacerbate wheezing. (Med 4o)
*nsubj-v*	Subject - Verb	Minimizing expenses is the best thing you can do. (Fin)	They may be able to provide more information about the application and help you understand what happened. (Fin 3.5 API).
*nsubj-v-advcl*	Subject - Verb - Adverbial Clause	Let me know if I can assist you further. (Med)	It sounds like you may be experiencing symptoms of depression and anxiety. (Med 3.5 API)
*nsubj-v-ccomp*	Subject - Verb - Clausal Complement	One of the fundamentals of technical analysis suggests that holding a security overnight represents a huge commitment. (Fin)	**This means people can buy** fewer goods and services with the same amount of money as before, effectively feeling poorer even though their nominal income hasn’t changed. (Fin 4)
*nsubj-vcop-acomp*	Subject - Copula Verb - Adjective Complement	Don’t worry, **you will be alright**. (Med)	However, **it is important** to consult a healthcare provider for an accurate diagnosis. (Med 4)
*nsubj-vcop-ncomp*	Subject - Copula Verb - Nominal Complement	**It’s a hassle**, but probably worth it just to recoup those funds. (Fin)	I**t’s always a good idea** to consult with a tax professional to ensure you are following the current rules and maximizing your tax benefits appropriately. (Fin 4)
*nsubj-v-dobj*	Subject - Verb - Direct Object	I understand your concerns. (Med)	**They** can **provide** personalized **advice** based on your financial situation and goals. (Fin 3.5 API)
*nsubj-v-xcomp*	Subject - Verb - Open Clausal Complement	The last two **questions seem to be asking** if you should buy MF or buy stocks directly. (Fin)	Estimated Taxes: As a self-employed artist, **you** may **need** to **pay** estimated taxes quarterly to cover income tax. (Fin 4o)
*v*	Verb	Having a good physical examination should help assess the cause of headache and medication **based** on it. (Med)	They can offer personalized treatment options **based** on your specific needs. (Med 4o)
*v-ccomp*	Verb - Clausal Complement	Making sure that the money that will trade is already there **makes the markets run smoothly**. (Fin)	It’s important to **note** that the SIPC does not **cover** losses due to market fluctuations or investment decisions. (Fin 3.5 API)
*v-dobj*	Verb - Direct Object	After 48 hours start doing warm saline gargles. (Med)	If your partner has gallstones, the doctor may recommend dietary changes to reduce the risk of further gallstones forming. (Med 3.5 Web)
*v-dobj-dobj*	Verb - Direct Object - Direct Object	It is unlikely to cause sweating or flashes. (Med)	Avoiding sugary foods and beverages is important to prevent blood sugar spikes. (Med 3.5 API
*v-xcomp*	Verb - Open Clausal Complement	You can indeed **create a rule set to make buy and sell decisions** based on the price action of your chosen security. (Fin)	It is typically considered a foundational principle in investing for most **seeking to balance** risk and return. (Fin 4)

*Note: This VAC was extremely rare in the AI-generated sub-corpora

**Table 6:** Financial domain Verb-Argument construction ranks and percentages across subcorpora.

	Human		3.5 Web		3.5 API		4 API		4o API	
	Construction	%	Construction	%	Construction	%	Construction	%	Construction	%
**1**	v-dobj	6.9%	v-dobj	10.0%	v-dobj	11.9%	v-dobj	11.5%	v-dobj	12.7%
**2**	nsubj-vcop-acomp	4.1%	nsubj-vcop-acomp	5.8%	nsubj-vcop-acomp	5.2%	nsubj-v-dobj	4.6%	nsubj-v-dobj	5.1%
**3**	nsubj-v-dobj	3.7%	nsubj-vcop-ncomp	4.4%	nsubj-v-dobj	3.8%	nsubj-vcop-acomp	4.2%	nsubj-vcop-acomp	3.8%
**4**	nsubj-vcop-ncomp	3.6%	nsubj-v-dobj	3.6%	v	2.1%	v	2.1%	v	2.2%
**5**	m-nsubj-v-dobj	1.7%	v-ccomp	2.1%	v-ccomp	2.1%	nsubj-vcop-ncomp	2.0%	v-ccomp	2.0%
**6**	nsubj-v-ccomp	1.6%	mark-nsubj-vcop-acomp	1.7%	nsubj-vcop-ncomp	1.9%	v-ccomp	1.9%	nsubj-vcop-ncomp	1.8%
**7**	v-ccomp	1.5%	mark-nsubj-v-dobj	1.7%	v-xcomp	1.4%	m-nsubj-v-dobj	1.3%	m-nsubj-v-dobj	1.4%
**8**	nsubj-v-xcomp	1.5%	nsubj-v-xcomp	1.4%	m-nsubj-vcop-acomp	1.3%	v-dobj-dobj	1.2%	v-dobj-dobj	1.3%
**9**	m-nsubj-vcop-acomp	1.4%	v	1.2%	m-nsubj-v-dobj	1.3%	m-nsubj-vcop-acomp	1.2%	nsubj-v-xcomp	1.2%
**10**	nsubj-v	1.3%	nsubj-v-ccomp	1.1%	v-dobj-conj_and	1.2%	nsubj-v-xcomp	1.1%	nsubj-v-ccomp	1.1%

**Table 7:** Medical domain Verb-Argument construction ranks and percentages across subcorpora.

	Human		3.5 Web		3.5 API		4 API		4o API	
	Construction	%	Construction	%	Construction	%	Construction	%	Construction	%
**1**	v-dobj	8.4%	nsubj-vcop-acomp	12.6%	v-dobj	12.8%	v-dobj	10.6%	v-dobj	11.6%
**2**	nsubj-v-dobj	5.5%	v-dobj	11.7%	nsubj-vcop-acomp	9.5%	nsubj-vcop-acomp	7.4%	nsubj-vcop-acomp	6.6%
**3**	nsubj-vcop-acomp	4.7%	nsubj-v-dobj	3.6%	nsubj-v-dobj	3.7%	nsubj-v-dobj	4.4%	nsubj-v-dobj	5.3%
**4**	nsubj-vcop-ncomp	3.6%	nsubj-vcop-ncomp	3.4%	v-ccomp	2.5%	nsubj-vcop-ncomp	2.2%	nsubj-vcop-ncomp	2.8%
**5**	v-ccomp	2.9%	v-dobj-conj_and	3.2%	v-dobj-conj_and	2.4%	v-ccomp	2.0%	v-ccomp	2.1%
**6**	nsubj-v-ccomp	2.0%	v-ccomp	2.7%	nsubj-vcop-ncomp	2.2%	nsubj-v	1.8%	m-nsubj-v-dobj	1.7%
**7**	m-nsubj-v-dobj	2.0%	m-nsubj-v-dobj	2.1%	v-dobj-dobj	2.1%	v-dobj-dobj	1.8%	nsubj-v-ccomp	1.7%
**8**	nsubj-v-advcl	1.6%	v-dobj-dobj	1.8%	m-nsubj-v-dobj	1.6%	m-nsubj-vcop-acomp	1.6%	nsubj-v	1.6%
**9**	nsubj-v-xcomp	1.5%	m-nsubj-vcop-acomp	1.5%	nsubj-v	1.6%	m-nsubj-v-dobj	1.5%	v-dobj-dobj	1.6%
**10**	dep-nsubj-v-dobj	1.4%	nsubj-v	1.3%	v	1.5%	v	1.4%	m-nsubj-vcop-acomp	1.6%

Considering the number of diverse VACs present in the dataset, it is striking that only 16 unique types account for the top ten VACs in all ten subcorpora (100 possible VAC slots) and that six VACs occur in all ten lists (*v-dobj, nsubj-vcop-acomp, nsubj-v-dobj, nsub-vcop-ncomp, m-nsubj-v-dobj,* and *v-ccomp*). Among these, transitive VACs, where an action is directed toward and affects the object, (*v-dobj* and *nsubj-v-dobj*) surface as key structures across contexts, with *v-dobj* occupying the number one slot in all subcorpora except the 3.5 Web Medical corpus. In the 3.5 Web Medical corpus the number one slot is *nsubj-vcop-acomp* and 45.8% of this are uses of the form “it is important” or “it’s important”, many of which direct the user to a medical professional. In the human-to-human comparison across Medical and Financial domains of discourse, only two top ten VACs are not shared across lists, although the relative importance varies, with somewhat more variation observable in the human-to-GPT comparisons and across GPT versions. Nevertheless, the consistency and pervasiveness of these VACs points to the communicative value of these linguistic structures and their meaning potentials across discourse, the extent to which AI-generated text broadly resembles human writing, and the usefulness of a VAC perspective for examining structural resources in linguistic data.

**Fig 1:**
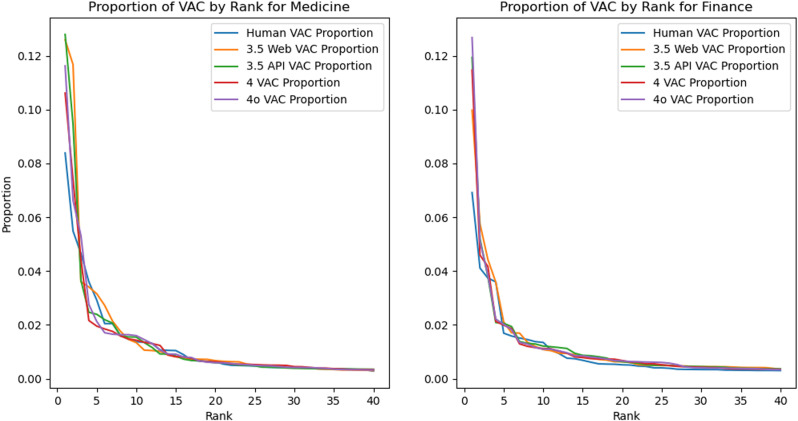
Proportions of VAC by rank.

Examining the VAC distributions more closely (see also Fig 1), the greater reliance on top VACs in AI-generated responses is striking. While human responses top out at 6.9% (Finance) and 8.4% (Medicine) of discourse represented by the top VAC (*v-dobj*), the top construction with AI-generated data represents over 10.6% of all verbal constructions (and over 12.6% in three cases). Examination of these figures also reveals that the VAC frequencies in AI-generated approximate the distributions in human-written text by around rank ten, with Finance beginning to align somewhat earlier than Medicine. This means that while AI-generated text makes use of largely the same VAC resources as humans do in both contexts, they tend to rely more heavily on a small number of highly prototypical forms. Once more, this reinforces the idea that the text generated by LLM-powered chatbots structurally resembles human writing, even in the relative importance of schematic constructions such as VACs, but it is different in measurable and meaningful ways.

We note that these comparisons are made without respect to any normalizations for the vastly different lengths of text. At the sample size and observed proportions of the top VACs these are insensitive to corpus size (the standard error is estimated from above by $\frac{1}{2\;\sqrt{n}}$ Investigating these observations, while important and interesting, is beyond the scope of this paper.

Two comparisons of the VACs themselves are made here in brief: across domains of discourse (Medicine and Finance) and models/interfaces. The human-written text demonstrates a relative consistency in the VACs used across Medical and Financial domains, with eight of ten VACs appearing in both lists with differences in the frequency ranks and percent of overall verbal constructions they represent. One key difference is that the transitive constructions (*v-dobj* and *nsubj-v-dobj*) account for a notably larger percent of overall VACs in Medical domains than they do in Finance (perhaps due to the physical nature of actions and entities in medicine, in comparison to the abstraction inherent to financial concepts), with some of the other constructions representing nearly identical percentages. In contrast, the transitive constructions are markedly more common in GPT-produced discourse, with a high reliance on the transitive *v-dobj* construction. And while the VACs in ChatGPT 3.5 Web present more consistent frequency ranks across discourse domains, there is clearly more domain-based variation in the corpora produced by the other GPT conditions than in the human-written text. Across underlying LLMs and interfaces (and as compared to humans), two key observations are present at the broad VAC level. One is that, as previously stated, AI-generated text shows abundant presence of a similar set of core verbal structures present in human texts, albeit with notably greater density of many such structures and some key differences. For example, many of the subcorpora of AI-generated text contain the *v* construction commonly, which is relatively infrequent in human discourse overall. Also, the *nsubj-v-ccomp* VAC, which is ranked sixth in human-written text for both domains, only occurs in the top ten with the most recent 4o model. This relates to the second key observation: text produced by ChatGPT models differs from each other in frequency ranks and relative distributions at the top.

### Verbs in Verb-Argument constructions across corpora

The verbs which occur within each VAC were also identified and compared across AI-generated and human-written text. Interestingly, the clear pattern of ChatGPT’s overreliance on top structures, which are nonetheless similar to structures in human-produced discourse, is not as consistent in the verb-type profile, with more VAC-specific patterns surfacing. Some, such as the VACs examined from a verb-frequency perspective in Tables 8 and 9, demonstrate small and variable differences across AI-generated and human-written text subcorpora comparisons, while others, such as those examined from a verb-frequency perspective in Tables 10 and 11, show marked disparities.

**Table 8:** Lemmatized verbs in finance v-dobj verb-argument construction by subcorpus.

	Human		3.5 Web		3.5 API		4 API		4o API	
	Verb	%	Verb	%	Verb	%	Verb	%	Verb	%
**1**	have	4.5%	make	11.0%	make	7.3%	make	4.0%	make	3.6%
**2**	make	4.5%	use	3.7%	consider-	3.8%	use	3.7%	understand-	3.3%
**3**	pay	4.1%	have	3.2%	have	3.0%	understand-	3.1%	use	3.2%
**4**	use	4.1%	find	2.6%	use	2.9%	consider-	2.9%	consider-	2.5%
**5**	buy	3.8%	pay	2.5%	determine-	2.6%	have	2.3%	ensure-	2.4%
**6**	do*	3.8%	buy	2.5%	provide-	2.2%	buy	2.0%	provide-	2.2%
**7**	get	3.6%	determine-	2.5%	reduce	2.2%	manage-	1.9%	have	2.0%
**8**	sell	1.9%	consider-	2.4%	understand-	2.0%	provide-	1.9%	maintain-	1.9%
**9**	find	1.6%	sell	2.2%	ensure-	2.0%	maintain-	1.9%	manage-	1.8%
**10**	take*	1.6%	get	1.9%	buy	1.9%	reduce	1.8%	avoid	1.8%
**11**	see*	1.3%	provide-	1.9%	seek*	1.8%	ensure-	1.8%	sell	1.7%
**12**	keep	1.2%	reduce	1.4%	sell	1.7%	sell	1.8%	reduce	1.7%
**13**	reduce	1.0%	purchase*	1.3%	find	1.7%	avoid	1.7%	buy	1.6%
**14**	cover	0.9%	manage-	1.3%	avoid	1.6%	keep	1.5%	get	1.5%
**15**	avoid	0.9%	cover	1.2%	create*	1.5%	pay	1.5%	keep	1.4%

Note: * indicates a verb unique to the subcorpus top 15; - indicates a verb unique to the GPT corpora

**Table 9:** Lemmatized verbs in medicine *v-dobj* verb-argument construction by subcorpus.

	Human		3.5 Web		3.5 API		4 API		4o API	
	Verb	%	Verb	%	Verb	%	Verb	%	Verb	%
**1**	take	8.0%	determine-	8.0%	determine-	5.3%	manage-	5.2%	reduce-	6.1%
**2**	use	6.9%	reduce-	5.4%	seek-	4.8%	reduce-	4.6%	manage-	5.3%
**3**	have	4.8%	recommend*	4.2%	reduce-	4.5%	avoid	3.8%	consult	4.6%
**4**	ask*	4.3%	see	4.0%	prevent	4.1%	seek-	3.7%	avoid	4.3%
**5**	get	3.9%	manage-	3.6%	manage-	4.1%	consult	3.5%	seek-	3.5%
**6**	answer*	3.8%	improve-	3.4%	improve-	3.6%	prevent	2.8%	improve-	2.9%
**7**	consult	3.6%	take	3.2%	avoid	3.5%	use	2.7%	take	2.8%
**8**	avoid	3.3%	treat	3.1%	address*	3.2%	keep-	2.5%	get	2.8%
**9**	do*	2.4%	prevent	3.1%	follow-	2.5%	consider*	2.4%	prevent	2.6%
**10**	cause*	2.2%	avoid	3.0%	have	2.5%	determine-	2.3%	make*	2.2%
**11**	help*	2.1%	follow-	2.9%	alleviate*	2.4%	improve-	2.3%	keep-	2.2%
**12**	prevent	1.8%	have	2.9%	provide*	2.4%	maintain-	2.2%	determine-	2.1%
**13**	contact*	1.4%	get	2.5%	take	2.4%	ensure*	2.1%	use	2.0%
**14**	see	1.2%	receive*	2.2%	consult	2.2%	have	2.1%	maintain-	1.9%
**15**	treat	1.2%	use	2.2%	concern*	1.8%	get	2.0%	have	1.8%

Note: * indicates a verb unique to the subcorpus top 15; - indicates a verb unique to the GPT corpora

**Table 10:** Lemmatized verbs in finance *v-ccomp* VAC across subcorpus.

	Human		3.5 Web		3.5 API		4 API		4o API	
	Verb	%	Verb	%	Verb	%	Verb	%	Verb	%
**1**	let	7.1%	note	26.0%	ensure-	28.5%	ensure-	28.2%	ensure-	33.9%
**2**	assume	6.2%	help	17.4%	help	17.5%	help	13.3%	help	16.0%
**3**	note	5.9%	ensure-	12.7%	note	14.8%	consider	5.5%	mean	4.6%
**4**	say*	5.2%	determine-	6.1%	determine-	4.2%	understand-	5.2%	understand-	4.0%
**5**	be	4.4%	let	3.8%	remember-	3.9%	decide-	4.6%	note	3.7%
**6**	see	3.9%	decide-	3.6%	understand-	3.7%	mean	3.7%	determine-	3.5%
**7**	know	3.9%	be	2.4%	consider	3.2%	determine-	3.6%	decide-	3.2%
**8**	do*	3.6%	consider	2.4%	mean	3.0%	note	2.9%	consider	2.4%
**9**	help	3.0%	understand-	2.2%	decide-	2.5%	see	2.0%	assume	1.4%
**10**	have	2.8%	see	2.0%	have	1.3%	remember-	1.5%	let	1.4%
**11**	get*	2.8%	mean	1.7%	see	1.3%	make	1.4%	see	1.3%
**12**	make	2.2%	have	1.6%	please*	1.2%	know	1.3%	suppose*	1.0%
**13**	mean	1.9%	make	1.2%	keep*	1.1%	assess-	1.2%	provide*	0.9%
**14**	consider	1.8%	plan*	1.0%	let	1.0%	evaluate*	1.1%	assess-	0.9%
**15**	show*	1.7%	remember-	0.9%	calculate*	0.8%	assume	1.0%	know	0.9%

Note: * indicates a verb unique to the subcorpus top 15; - indicates a verb unique to the GPT corpora

**Table 11:** Lemmatized verbs in medicine *v-ccomp* VAC across subcorpus.

	Human		3.5 Web		3.5 API		4 API		4o API	
	Verb	%	Verb	%	Verb	%	Verb	%	Verb	%
**1**	let	51.2%	help	51.1%	help	43.4%	ensure-	26.8%	ensure-	28.4%
**2**	please	8.3%	note-	16.9%	ensure-	12.3%	help	24.9%	help	20.1%
**3**	get	5.6%	hear-	7.5%	hear-	10.7%	hear-	7.3%	hear-	17.4%
**4**	help	3.6%	have	4.7%	note-	9.2%	please	6.1%	give-	5.9%
**5**	hope*	2.7%	ensure-	4.5%	remember-	6.0%	remember-	3.4%	please	3.1%
**6**	know	2.1%	remember-	3.1%	have	3.6%	note-	2.9%	remember-	2.5%
**7**	say	2.1%	let	1.7%	give-	2.5%	keep-	2.4%	note-	1.9%
**8**	ask*	1.5%	determine-	1.5%	please	1.9%	give-	2.3%	have	1.5%
**9**	have	1.2%	keep-	1.2%	keep-	1.8%	have	1.9%	keep-	1.5%
**10**	do*	1.2%	say	0.9%	let	0.8%	understand-	1.9%	let	1.4%
**11**	make	1.2%	suggest-	0.9%	suggest-	0.6%	consider-	1.6%	get	1.2%
**12**	wish*	0.9%	get	0.7%	result*	0.5%	here*	1.1%	consider-	1.1%
**13**	start*	0.6%	predict*	0.5%	know	0.4%	see*	1.0%	determine-	1.0%
**14**	confirm*	0.6%	rest*	0.4%	understand-	0.4%	make	1.0%	suggest-	1.0%
**15**	mean	0.6%	mention*	0.4%	mean	0.4%	suggest-	1.0%	understand-	0.8%

Note: * represents verb unique to subcorpus; - represents verb unique to GPT; Also note the presence of *here* in the 4 API subcorpus, as in the example “**Heres** a plan that might be considered…” (emphasis added). This refers exclusively to the use of *heres* with no apostrophe, and while it may be considered a tagging error, it appears in the human and 4 API subcorpora in similar patterns.

### Lemmatized verbs in *v-dobj*

Tables 8 and 9 present the top 15 verb types for the most prevalent VAC overall: *v-dobj*. A cross-domain examination highlights that, with the notable exception of ChatGPT 3.5 web, the verbs which most commonly communicate transitivity in *v-dobj* vary by domain. Restricting ourselves to VACs in human writing, only six of the top 15 verbs are common in the Finance and Medicine domains, roughly consistent with the AI-generated text conditions.

AI-generated text in the Finance domain makes use of roughly two-thirds of the same verbs in the top portion of the frequency lists for this VAC, but a few interesting differences are present. First, verbs “have,” “pay,” and “do” are not nearly as frequent in AI-generated text for this domain. Second, it appears that ChatGPT and humans tend to place verbs with different semantic connotations in this transitive construction. The more frequent verbs in AI-generated text appear to be more associated with analysis and reflection (e.g., “determine,” “consider”) or managing behaviors (e.g., “improve,” “follow,” “manage,” “ensure,” “avoid”). ChatGPT 3.5 web, and to a lesser extent its API homologue, present many verbs that uniquely appear in the top 15 verbs, but both 4 and 4o rely on six of the top fifteen verbs, each of which are less common in human discourse. We note that the verb “make”, which is the most frequent verb in *v-dobj* for all the GPT Finance subcorpora and the second most frequent in human text, shows some strong distinctions. Of the 3500 uses of *make* in AI-generated text, 571 of them are “before making any,” 338 are “making a decision”, and 245 are “make informed,” whereas these same patterns cover only three out of 301 instances of “make” in human-written text. Contextually, these all place conditions on decision-making and serve a hedging and distancing function.

The lemmatized verbs used in the *v-dobj* VACin AI-generated text in the Medical domain are notably distinct from human discourse in terms of the transitive verbs used. The top 6 verbs in the human corpus are either not used in the top 15 or are used drastically less in the all of the AI-generated text subcorpora (e.g., “take” is found in 8% of the VACs in human writing vs. roughly 1/3–1/4 as often in VACs in all AI-generated text), and other key frequency differences are found throughout. We see 14.1% of the uses of “take” in human text being “take care regards” and “ok and take care,” which frame the advice within a human interaction. These same constructions never appear in AI-generated text. On the other hand, we see 8.1% of AI-generated text being “before taking any” which once more places conditions on advice. This construction does not appear in human text. Within *v-dobj* the lemmatized verb “consult” is used 35 times in human text vs. 714 times in AI-generated text. Out of those 714 occurrences, 368 (51.5%) occur in the phrase “consult a healthcare [profession word]” which never appears in human-written text. Humans similarly recommended seeking the care of specialists, but in dynamic and locally responsive ways (e.g., “Honestly speaking, this is serious health issue and this can worsen with the age. So consult cardiologist as soon as possible for this”), rather than through formulaic language. A further 77 of the 714 instances are covered by either “consult your healthcare” or “consult a specialist”, which again never appear in human-written text. Similar to the AI-generated vs. human-written text comparisons in Finance, the GPT models show a reliance on more analytical or managerial verbs than those related to actions. This is reflected not only in ChatGPT’s lower interactional framing and lower provision of direct advice, but also in ChatGPT’s frequent hedging and distancing of advice through discussion of the processes of decision-making, rather than concrete verbs of action.

Considering the top VAC in the corpus overall, the findings indicate that AI-generated texts are generated using many of the same macro-level VAC resources as those that humans used, and these VACs construe many similar actions and meanings. However, the key differences in the types of meanings and relationships represented in AI-generated texts suggest important divergences in the way that ChatGPT provides advice and presents information.

### Lemmatized verbs in *v-ccomp*

This is further evidenced by an examination of the *v-ccomp* VAC. This VAC appeared in a position between 4 and 7 in all ten subcorpora, indicating a remarkable consistency in the importance of this schematic pattern. However, the top verbs in AI-generated texts do not resemble those in human-written texts in either Medical or Financial domains. In human writing in the Medicine domain, the lemmatized verb “let” represents 51.2% of all occurrences of the *v-ccomp* VAC, with the trigram “let me know” accounting for 98.8% of all of those occurrences. In human writing in the Finance domain, “let” represents 7.1% of the verbs that occur in this VAC. However, it is relatively rare (a maximum of 1.7%) in all other subcorpora except ChatGPT 3.5 Web Finance, where it accounts for 3.8% of the lemmatized verbs occurring in *v-ccomp*. In contrast, the verbs “help” and “ensure” occupy a combined 41.5% to 55.7% of all instances of *v-ccomp* in all the AI-generated text subcorpora except ChatGPT 3.5 Web where they together account for only 30.1%., but only 3.9% of human-written instances of *v-ccomp* in Medicine. and 4.5% of human-written instances of *v-ccomp* in Finance. Interestingly, while ChatGPT is positioned and marketed as a chatbot, the abundant use of “let me know” reflects a more overt dialogic and and ongoing interaction in human discourse that contrasts the more monologic tone which is pervasive in GPT-generated text and reinforced by GPT’s verbosity.

In Medicine, the trigrams “to help manage”, “to help reduce”, and “to help alleviate” account for 43% of ChatGPT uses of the word *help* in the *v-ccomp* VAC. These same collocations never occur in the human-written text, where a third of the occurrences are “to help you.” The pattern in the Finance domain is not quite as stark, but 20.8% of all uses of *help* by GPT (or 412 times out of 1,982 uses of help) are covered by the “to help reduce,” “to help manage,” “considerations to help you make,” “to help alleviate,” “to help determine,” and “considerations to help you decide”. None of these appear in the human-written text.

### Usage of nsubj-vcop-acomp

Even in VACs that afford little room for variation in verbs, such as those which are copula-be based, which refers to the use of ‘be’ to connect a subject and a complement, a usage-pattern analysis reveals key differences in how even key schematic meaning-making resources are employed. For example, *nsubj-vcop-acomp* occurs in the top three VACs of all subcorpora nearly exclusively with the lemmatized verb “be”, but ConcordanceCompass reveals key linguistic differences. In the Medicine domain, 30.5% of all uses of the copula be in *nsubj-vcop-acomp* in AI-generated texts were either “it is important” or “it’s important” whereas for human written texts these covered only 1.1% of the uses of the copula be in *nsubj-vcop-acomp*. Of the AI-generated “it is important” or “it’s important” sentences, 36.3% of the uses are covered by “important to speak”, “important to seek”, “important to see”, “important to consult”, or “important to discuss”, which are all directions to seek medical expertise, but these never appear in the human written text. However, 10.6% of the uses of the copula be in *nsubj-vcop-acomp* by humans in the Medical domain are covered by “will be happy” with 53 of the 57 uses being either “I will be happy to help” or “I will be happy to answer”, which resonates with other findings of politeness and dialogic framing in human responses. ChatGPT’s uses of the copula be in *nsubj-vcop-acomp* in the Medical domain never contain “will be happy”.

### Discovering contextual comparisons

Even restricting to the level of a VAC and lemmatized verb pair, our corpus is large enough that there could be hundreds or even thousands (in the case of the copula verb) of sentences containing the pair. This makes manually examining the key words in context, a traditional approach to understanding word usage, problematic. In order to provide insight into the differences in how words are used between corpora, we have developed a tool called “ConcordanceCompass”, that compares the relative frequencies of contexts (windows around a target word) within the two corpora and ranks them according to a variant of the metric $log_{2}\frac{\pi_{\max}}{\pi_{\min}}$ which was introduced by Andrew Hardie as Log Ratio Score in a 2014 blog post. This metric can be derived as the difference in the Pointwise Mutual Information (PMI) between the indicators of context presence and the indicator of corpus membership. If a term is present in only one corpus then the metric is infinite. Hastie resolves this by assigning an absolute frequency of 0.5 to terms which do not appear. As with usual rankings involving PMI, this scoring tends to select rare terms. With PMI rankings, one possible solution is to use the PMI^k family of metrics but when maximizing the difference they lead to a scalar multiple of the existing score and do not affect the rankings. We adopt an approach that does two things:

1. Bounds that maximum ratio from above. If we have a term that is present in only one corpus then it is assigned this maximum ratio rather than infinity. In our case, $R_{\max}=5$.,
2. We multiply the ratio by the maximum proportion raised to a power ɑ, which is our case is 0.2.

This approach leads us to the modified metric

$log_{2}(R\cdot \pi_{\max}^{\alpha })$ where $R=min\left\{\frac{\pi_{\max}}{\pi_{\min}},\;R_{\max}\right\}$

This modified metric has the property that for a fixed ratio R more frequent contexts are preferred.

## Discussion

This study adopts a Usage-Based Construction Grammar perspective to compare human and AI generated medical and financial advice through the most common VACs, the most common verbs within them, and prominent phraseological patterns of local use. Our analysis demonstrates the potential richness of using a usage-based construction approach in comparisons between human-written and AI-generated text. Across GPT models and interfaces, the similarity in VAC ranks between ChatGPT and human writers suggests that the way ChatGPT produces language reflects constructions like VACs that humans draw on to communicate. This suggests that transformer-based LLMs not only contain construction-like representations, but also that they can reproduce them at scale on a variety of topics, at least within this genre. We also see more subtle similarities. For example, while telehealth queries tend to be longer, answers to finance questions contain more words and sentences for both ChatGPT and human experts. This topic domain effect does not hold for sentence length. Neither ChatGPT nor human experts tend to vary sentence length notably in their responses to medical and financial queries. We hypothesize that the broad genre of giving advice may impose requirements about sentence length that ChatGPT has captured from its training data, possibly as part of its mandate to “follow the user’s instructions helpfully and safely” [16]. Importantly, we also observe clear evidence that both humans (as has been widely demonstrated through genre and register analysis) and to some extent ChatGPT both vary their language to meet the demands of a communicative task.

We also observe both obvious and subtle differences in the language used by human writers and produced by ChatGPT. The most salient is verbosity. Leaving aside broad similarities in sentence length, ChatGPT produces significantly more words per response than human experts (perhaps becoming long-winded), with successive models growing ever more wordy (see Table 3). The VAC analysis shows a more subtle, though quite pervasive, difference. Although VAC ranks are broadly similar between human writers and ChatGPT, which suggests that ChatGPT uses similar constructional resources as humans do, there are clear differences in the prominence of individual VACs within these ranks (see Tables 6 and 7). For one thing, ChatGPT’s distribution for top VACs is significantly more head-heavy. In the Finance domain, the most common VAC in human-written text accounts (*v-dobj*) accounts for 6.9% of all VACs, compared to ChatGPT’s 10% (3.5 Web), 11.9% (3.5 API), 11.5% (4 Web) and 12.7% (4o Web) a trend which becomes even more significant when we normalize for differences in text length. The top VAC in text written by telehealth experts (*v-dobj*) accounts for 8.4% of all VACs vs. ChatGPT’s 12.6% (3.5 Web), 12.8% (3.5 API), 10.6% (4 Web) and 11.6% (4o Web). This reliance on prototypical construction patterns may result from the requirement that ChatGPT’s output be simple and clear. That goal may translate to a narrow set of rhetorical stances compared to telehealth providers and financial advisers, resulting in a preference for a smaller array of core meaning-making resources. Highly frequent, prototypical constructional resources are hypothesized to reoccur and be reinstantiated because they are entrenched in the minds of language users and afford important and recognizable communicative functions [35,41–43], which also supports clarity in ChatGPT produced language.

More closely examining specific VACs and related verbs fleshes out these differences more starkly. For example, in ChatGPT with GPT3.5 Web’s medical responses, provisos like “it is important to consult a healthcare provider for…” are so frequent that the *nsubj-vcop-acomp* VAC is the top-ranked VAC, surpassing the most common VAC in every other sub-corpus (the transitive *v-dobj*). In stark tonal contrast, a common production of the same VAC in human medical advice is “Don’t worry, you will be alright.” Intriguingly, this pattern did not extend to the Finance domain where *nsubj-vcop-acomp* only accounted for 5.8% of all VACs, compared to 12.6% for Medicine. This pattern did not hold when the same medical queries were submitted to subsequent models: ChatGPT 3.5 API (9.5%), 4 API (7.4%) and 4o API (6.6%). It is possible that the proliferation of this pattern was picked up on my human annotators at some point, resulting in efforts to curb this behavior in subsequent models. Regardless, it is a clear example of an important pattern. ChatGPT often produces language which contains important VAC resources of human language in its own language production, although with distinct meanings and forms. It would be unscientific and personifying to call this voice, but similar behavior by an agentive language user could be discussed as such.

In the two VACs examined in detail, whereas humans tend to use shorter lemmatized verbs with Germanic roots (e.g., “have,” “make,” “buy,” “do,” “find,” “get”), ChatGPT selects for longer verbs with Latinate origins (e.g., “determine,” “manage,” “reduce,” “consult,” “consider,” “provide,” “ensure”). ChatGPT’s use of these verbs may suggest avoidance of more informal language more broadly, and it also underscores the complexity of categorizing telehealth interactions as a genre practice, as humans showed regular use of a variety of speech-like features in written form. Examination of the verbs used also capture that ChatGPT’s advice tends to be more indirect, focusing on planning and other cognitive processes, compared to human experts’ preference for direct, action-oriented feedback. Our keyword-in-context analysis provides examples of these tendencies and broader insights into the nature of the differences in purpose and framing that may account for them.

For instance, in the most common VAC, (*v-dobj*), “pay” is the third most common verb used by financial advisors, but virtually non-existent in AI-generated text. On the other hand, we saw ChatGPT directing participants to ‘manage’ and ‘consider’ problems, while human experts focused more directly on what managing and resolving a problem may look like. In medical queries, the verb “consult” appeared in AI-generated text in *v-dobj* more than 20 times as often as in human-written text, mostly to advise users to “consult a healthcare professional”. Considering the implications of this more broadly, telehealth professionals, logically, do not typically need to tell patients to consult a healthcare professional as they are already doing just that. At the same time, they provide considerable dialogic framing to these interactions, which reflects broader conventions of in-person human-human interactions even in a quick written exchange. For instance, in *v-ccomp,* the most frequent human verb is ‘let’, most commonly in phrases like “let me know,” which invite follow-up. This expression is nearly absent from all ChatGPT language across models. Likewise, while the verb ‘help’ is common in this VAC across subcorpora, humans tend to use *“to help you [verb]”,* which does not occur a single time in ChatGPT in this VAC.

## Conclusion

Overall, we believe that this study points towards the considerable potential for utilizing Usage-Based lenses, and in particular Construction Grammar for analyzing ChatGPT language production, as well as other forms of AI-generated text. Verb-Argument Constructions proved to be a meaningful point of linguistic comparison, both at the construction level and at the level of verb distribution internally. VACs encapsulate not only conventionalized structural relationships, but also semantic and pragmatic potentials that shape discourse. While it is clear that there is considerable structural similarity across humans and ChatGPT, we also see that the AI-generated texts in these corpora exhibit markedly different semantic and pragmatic goals than those we observe in human written texts that use the same resource. The VAC analysis allows us to collect data points in an empirically robust manner that highlights similarities and these important differences. Moving beyond whether or not human-written and AI-produced text are distinguishable allows us to see how they accomplish similar, but critically different rhetorical ends. By combining VAC and Key Word in Context analysis, researchers can see at scale the recurrent meanings being expressed across texts in the sub-corpora.

In a sense, this suggests that ChatGPT successfully leverages its training data and utilizes pattern-data that resembles constructions to accomplish goals aligned with the product. Despite the use of the word *chat* in the name, the communicative purpose of these ChatGPT systems when prompted with a short-form question is to provide a long-form informative answer, and not to initiate a chat. In our corpora, faced with identical questions, humans typically gave shorter answers using less formal language, and frequently left rhetorical space for follow on communication and more overt chat framing. We notice that the tendency for lengthy and more elaborated answers increases with successive models.

Building on the findings of this research, we think that considerable future opportunities exist to employ a VAC perspective to examining ChatGPT and other AI-generated Text. In particular, while we focused on the most prototypical patterns and the consistency and variation within those patterns, other research can adopt more statistically-driven comparisons of VAC use overall or otherwise examine creativity more directly. Likewise, while our comparison of two topic domains, medical and financial, highlighted important variation in both human and ChatGPT language, future research can more directly investigate the effects of topic and, perhaps more importantly, register and genre, to examine if ChatGPT varies language production as much as humans do in relationship to their communicative situation. As a final recommendation, we call for future research which adopts a VAC perspective to non-English text.
